# A large-scale study of chronic sleep disorders in psychiatric inpatients: Prevalence, hospitalization burden, restraint use, and comorbidities

**DOI:** 10.1192/j.eurpsy.2025.10131

**Published:** 2025-11-19

**Authors:** Pierre Alexis Geoffroy, Romain Roure, Sophie B. Sebille, Julia Maruani, Estelle Taupinard, Michel Lejoyeux, Anne Perozziello, Sibylle Mauries

**Affiliations:** 1 Département de psychiatrie et d’addictologie, AP-HP, GHU Paris Nord, DMU Neurosciences, Hopital Bichat – Claude-Bernard, F-75018 Paris, France; 2 Université Paris Cité, NeuroDiderot, Inserm, F-75019 Paris, France; 3 Centre Chronos, GHU Paris - Psychiatry & Neurosciences, 1 rue Cabanis, 75014 Paris, France; 4 CNRS UPR 3212, Institute for Cellular and Integrative Neurosciences, F-67000 Strasbourg, France; 5 Direction de l’Innovation Technologique et du Système d’Information, GHU Paris psychiatrie & neurosciences, 1 rue Cabanis, 75014 Paris, France; 6 Cellule Epidemiologie, GHU Paris psychiatrie & neurosciences, 1 rue Cabanis, 75014 Paris, France

**Keywords:** bipolar disorder, depression, hypersomnia, insomnia, parasomnia, psychiatric disorder, schizophrenia, sleep disorder

## Abstract

**Background:**

Sleep disorders are closely linked to the onset, progression and severity of psychiatric disorders, yet large-scale data from real-world inpatient settings remain limited. Evaluating the impact of chronic sleep disorders (CSD) in this context is essential for improving care.

**Methods:**

We conducted an analysis of adult inpatients hospitalized from January 1, 2021, to December 31, 2023, using data from the Paris Psychiatry Hospital Group’s health data warehouse. Sleep disorders were identified via ICD-10 codes, hypnotic prescriptions, or mentions in medical record. CSD was defined using an Index of Length of Stays with Disorders (ILSD) >0.5, and no sleep disorders (NSD) with an ILSD of zero.

**Results:**

Among 13,913 psychiatric inpatients, 81% were classified as having CSD. Compared to NSD patients, those with CSD had a higher number of hospitalizations (1.84 vs 1.33, p<0.001) and increased use of seclusion (17.6% vs 13.3%, p<0.001) and physical restraint (6.6% vs 5.3%, p=0.003). Individuals with CSD were more frequently hospitalized than the NSD group for depressive disorders (15.6% vs13.1%, p<0.001), bipolar disorders (11.4% vs5.6%, p<0.001), personality disorders (5.3% vs4.3%, p=0.009), alcohol abuse (3.3% vs2.4%, p=0.005), other substance use disorders (2.9% vs2.2%, p=0.018), manic episode (2.0% vs0.9%, p<0.001), and anxiety disorders (1.4% vs0.9%, p=0.012). Hypnotics were prescribed in 50.5% of SD-related stays. The CSD group had more psychiatric and non-psychiatric comorbidities.

**Conclusions:**

CSD are highly prevalent in psychiatric inpatients and associated with more severe clinical profiles, greater hospitalization burden, and increased restraint use. Targeted sleep management strategies may help improve outcomes and care.

## Introduction

Sleep disorders are highly prevalent in psychiatric populations and are increasingly recognized as core dimensions rather than secondary symptoms of psychiatric disorders [1–4]. Research has demonstrated bidirectional relationships between sleep disorders and psychiatric disorders [5–7]. Additionally, sleep complaints – including insomnia, hypersomnia, sleepiness, and nightmares – have been shown to predict suicide attempts independently of any psychopathology [8]. These sleep disturbances can exacerbate psychiatric symptoms, increase comorbidities, and elevate relapse risk [6, 9]. Sleep disorders are also among the earliest prodromal signs of psychiatric conditions. In the Adolescent Brain and Cognitive Development study, which followed over 11,000 children longitudinally, sleep disturbances emerged as the strongest predictor of mental health risk in adolescence – outweighing even adverse childhood experiences and family history of psychiatric illness [10]. These alterations also appear to predict imminent mood relapse or recurrence. A key concept in understanding the progression of mood disorders is the “Chronos syndrome,” defined as “a clinical syndrome that reliably predicts an imminent transition to a mood episode” [11]. In a recent study, 87.5% of patients reported sleep–wake disturbances in the days and weeks preceding a depressive episode, and 76.5% before a manic episode – further supporting the predictive value of sleep disorders in mood disorders [12]. Despite growing evidence, sleep disorders in psychiatric patients remain largely underdiagnosed and undertreated, particularly in inpatient settings.

Previous studies have documented high rates of insomnia, hypersomnia, parasomnias, and circadian rhythm disorders across various psychiatric disorders, including bipolar disorder [13–17], major depressive disorder [7, 18, 19], schizophrenia [3, 20], suicide [21–25], and substance use disorders [26–30]. Most of this work has focused on outpatients, specific diagnostic categories, or relatively small samples. Large-scale studies in psychiatric inpatients have also demonstrated a high prevalence of insomnia and other sleep problems, but often without addressing the persistence of symptoms or their clinical consequences. For instance, Talih et al. [31] reported that 67.4% of hospitalized psychiatric patients (n = 203) screened positive for insomnia using the Insomnia Severity Index. Similarly, in a Dutch sample (n = 1082), Mijnster et al. [32] found that 46.2% of individuals with mental disorders scored above the cut-off for having a sleep disorder on the Holland Sleep Disorders Questionnaire. These studies, though informative, did not examine the longitudinal course of chronic sleep disorders (CSDs) or their broader implications across hospital stays [33]. To date, no work has explored their impact on hospitalization burden, restraint use, and comorbidities, despite their high clinical and public health relevance. Large-scale, real-world studies in psychiatric hospitals are therefore crucial to better understand the prevalence and consequences of CSDs in this vulnerable population.

In this context, we aimed to systematically assess the prevalence and clinical characteristics of CSDs – defined as sleep disturbances affecting the patient during at least half of their hospital stays and not merely episodic – in a large cohort of adult psychiatric inpatients. Specifically, we sought to: (i) compare hospitalization characteristics (length of stay, restraint use, admission type) between patients with CSD and those without sleep disorders (NSD); (ii) examine diagnostic differences, particularly regarding mood, psychotic, addictive, developmental, eating, and personality disorders; and (iii) assess hypnotic medication use and prescribing patterns across diagnostic categories.

By leveraging data from the health data warehouse of the Paris Psychiatry Hospital Group, we hypothesize that this study will provide novel insights into the real-world impact of CSDs in psychiatric inpatient care.

## Methods

### Setting and study population

This retrospective study used routinely collected data extracted from the health data warehouse of the Paris Psychiatry Hospital Group (GHU Paris Psychiatrie & Neurosciences). This data warehouse is a secure, centralized repository that consolidates routinely collected health data of around 500,000 psychiatric patients. This data warehouse records all hospitalization information: administrative information (age, gender, type of admission, etc.) and medical information (primary diagnosis – the reason why the patient has been admitted and comorbidities, i.e., complications and morbidities that impact the course of hospitalization). The data warehouse also includes biological results, drug prescriptions, textual medical and imaging reports. It serves as a vital resource for researchers and healthcare professionals, facilitating data-driven insights and advancements in psychiatry.

#### General definition of CSDs

CSDs (CSD) were identified using a combined approach: ICD-10 diagnostic codes, hypnotic prescriptions, and textual mentions in electronic medical records. Patients were considered to have CSD when sleep disturbances were documented in more than half of their hospitalizations, according to our predefined Index of Length of Stays with Disorders (ILSD). This operationalization was chosen to reflect the persistence of symptoms over time, rather than transient sleep problems limited to one admission. Sleep diagnoses were captured from the entire hospitalization record, not solely at admission, and thus included both preexisting disorders and those documented during hospitalization. Hypnotic use was extracted from prescriptions during hospitalization; information about treatments initiated prior to admission was not systematically available. While this approach does not allow precise differentiation between insomnia, hypersomnia, and parasomnia, it increases ecological validity by capturing the broad spectrum of chronic sleep disturbances encountered in psychiatric inpatient settings.

#### Detailed operationalization

In more detail, we included all adult patients (aged 18 years and older at the time of admission) with at least one full-time psychiatric hospitalization from January 1, 2021 to December 31, 2023. Stays were flagged for sleep disorders if patients met at least one of the following three criteria:

- Diagnosis related to sleep disorders based on the International Classification of Diseases (ICD-10) diagnosis codes: F51 and G47.
- Drugs administered for sleep disorders (hypnotics): “Stilnox, Zolpidem, Imovane, Zopiclone, Circadin, Melatonin, Slenyto, or Theralene.”
- Textual evidence of sleep-related terms (e.g., insomnia, nightmares, drowsiness) within unstructured medical documents.

**Figure 1.**
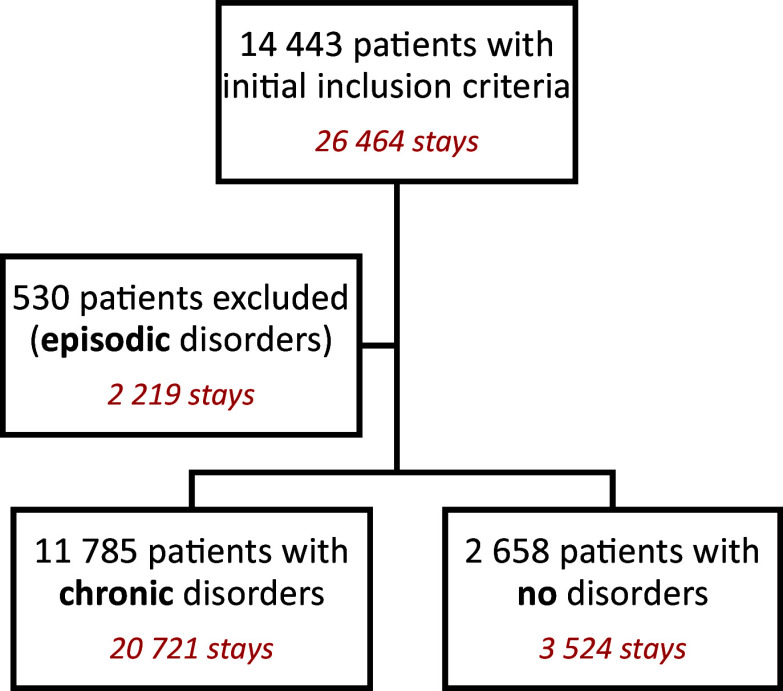
Study flow chart.

A total of 14,443 patients satisfying the criterion were included in this study (representing 26,464 stays). Among them, 11,785 patients presented with sleep disorders. The population with sleep disorders was finetuned by distinguishing CSDs from episodic ones. For that, we built a metric called the ILSD, defined as the ratio of the cumulative stay duration with sleep disorders to the cumulative duration of all stays over the study period. CSD patients corresponded to an ILSD greater than 0.5, whereas patients with no sleep disorders (NSDs) had an ILSD score of zero. Patients with episodic disorders were excluded from the analysis. The study flow chart is shown in Figure 1.

This study was approved by the Research Ethics Committee of the Paris Psychiatry Hospital Group (accreditation no. 2024-CER-D-004).

### Variables

All data were extracted from the GHU health data warehouse. We selected specific variables among patient characteristics (gender and age, number of stays per patient) and stay characteristics (admission and discharge dates, type of admissions [involuntary admission or not]), and restraint practices such as seclusion and/or physical restraint measures, administration of hypnotic medications, primary diagnosis, and comorbidities (based on the 10th revision of ICD-10 diagnosis codes). Diagnoses were categorized according to medical similarities as assessed by medical professionals.

### Statistical analysis

Quantitative variables were described using the mean and standard deviation. Qualitative variables were described as frequencies and percentages.

Comparisons between the NSD and CSD groups were conducted using Chi-square or Fisher’s exact tests for categorical variables and Student’s *t* tests for continuous variables, as appropriate.

All tests were two-sided, with a statistical significance level set at 0.05. All analyses were performed using Python (version 3.10.9) and Stata (version 18).

## Results

### Global analyses: Characteristics of patients and hospital stays

Our study included 13,913 patients, with 48.7% women and an average age of 41.2 years (Table 1). There were 2658 patients in the NSD group and 11,255 patients in the CSD group (CSDs), resulting in a CSD prevalence of 81% among hospitalized psychiatric patients. The proportion of women was significantly higher in the CSD group than in the NSD group (49.1% vs. 46.9%, p < 0.045). No significant age differences were observed between the two groups.

**Table 1.** Characteristics of patients and hospital stays

Patients’ characteristics	No sleep disorders (NSD) group (N = 2 658)		Chronic sleep disorders (CSD) group (N = 11 255)		All patients (N = 13 913)		
Sex	N	%	N	%	N	%	*p*-value
Men	1411	53.1%	5732	50.9%	7143	51.3%	**0.045**
Women	1247	46.9%	5523	49.1%	6770	48.7%	
**Age**	**Mean**	**SD**	**Mean**	**SD**	**Mean**	**SD**	*p* **-value**
All patients	41.3	16.4	41.2	16.6	41.2	16.6	0.8128
Men	40.3	14.9	39.8	15.3	39.9	15.3	0.2576
Women	42.3	17.9	42.6	17.7	42.6	17.7	0.6258
**Number of hospitalizations per patient**	1.33	1.99	1.84	2.30	1.74	2.25	**<0.001**

Stays characteristics	No sleep disorders (NSD) group (n = 3 524)		Chronic sleep disorders (CSD) group (n = 20 721)		All patients (n = 24 245)		
Admissions and restraint practices	N	%	N	%	N	%	*p*-value
Involuntary admission	2087	59.2%	12261	59.2%	14348	59.2%	0.955
Seclusion measure	467	13.3%	3642	17.6%	4109	16.9%	**<0.001**
Physical restraint measure	186	5.3%	1368	6.6%	1554	6.4%	**0.003**
**Duration**	**Mean**	**SD**	**Mean**	**SD**	**Mean**	**SD**	***p*-value**
Seclusion	8.85	13.01	13.53	23.73	13.00	22.82	**<0.001**
Physical restraint	4.73	6.15	5.46	9.44	5.37	9.11	0.3006

*Note:* The bold text represents the statistically significant results (*p*<0.05)

On average, patients in the NSD group had 1.33 hospital stays during the study period, compared to 1.84 stays in the CSD group (p < 0.001), leading to an analysis of 24,245 hospitalizations in total, with 3,524 stays in the NSD group (14.5%) and 20,721 in the CSD group (85.5%).

Over half of the stays were involuntary admissions (59.2%), with no difference between the groups. However, patients in the CSD group experienced seclusion more frequently during hospitalization than NSD patients (17.6% vs. 13.3%, p < 0.001), as well as physical restraint measures (6.6% vs. 5.3%, p = 0.003). In addition, the duration of seclusion was significantly longer for CSD patients (13.53 days) compared to NSD patients (8.85 days), while the length of physical restraint use did not differ between groups.

### Clinical description of hospital stays

Regarding admission motives, there was more hospitalizations in the CSD than in the NSD group related to depressive disorders (15.6% vs. 13.1%, p < 0.001), bipolar disorders (11.4% vs. 5.6%, p < 0.001), personality disorders (5.3% vs. 4.3%, p = 0.009), alcohol abuse (3.3% vs. 2.4%, p = 0.005), substance use disorders (other than alcohol and cannabis) (2.9% vs. 2.2%, p = 0.018), manic episode (2.0% vs. 0.9%, p < 0.001), and anxiety disorders (1.4% vs. 0.9%, p = 0.012). In reverse, there was significantly more stays in the NSD group than in the CSD group for schizophrenia or persistent delusional disorders (41.4% vs. 36.0%, p < 0.001), transient psychotic disorders (7.0% vs. 5.7%, p = 0.003), reaction to severe stress (4.2% vs. 3.2%, p = 0.002), developmental disorders (3.6% vs. 1.1%, p < 0.001), or eating disorders (2.1% vs. 0.9%, p < 0.001) (Table 2).

**Table 2.** Clinical description of hospital stays

	No sleep disorders (NSD) group (n = 3 524)		Chronic sleep disorders (CSD) group (n = 20 721)		All patients (n = 24 245)		
Main diagnosis	N	%	N	%	N	%	*p*-value
Schizophrenia and persistent delusional disorders	1460	41.4%	7468	36.0%	8928	36.8%	**<0.001**
Depressive disorders	460	13.1%	3224	15.6%	3684	15.2%	**<0.001**
Bipolar disorders	199	5.6%	2363	11.4%	2562	10.6%	**<0.001**
Acute transient psychotic disorders and other psychotic disorders	247	7.0%	1185	5.7%	1432	5.9%	**0.003**
Personality disorders	150	4.3%	1101	5.3%	1251	5.2%	**0.009**
Reaction to severe stress	149	4.2%	667	3.2%	816	3.4%	**0.002**
Disorders due to alcohol use	85	2.4%	685	3.3%	770	3.2%	**0.005**
Disorders due to other psychoactive substance use	78	2.2%	607	2.9%	685	2.8%	**0.018**
Other mood disorders	59	1.7%	510	2.5%	569	2.3%	**0.004**
Other psychiatric diagnosis	88	2.5%	365	1.8%	453	1.9%	**0.003**
Manic episode	33	0.9%	412	2.0%	445	1.8%	**<0.001**
Other nonpsychiatric diagnosis	64	1.8%	356	1.7%	420	1.7%	0.680
Developmental disorders	127	3.6%	229	1.1%	356	1.5%	**<0.001**
Anxiety disorders	31	0.9%	291	1.4%	322	1.3%	**0.012**
Disorders due to cannabis use	40	1.1%	277	1.3%	317	1.3%	0.330
Eating disorders	75	2.1%	179	0.9%	254	1.0%	**<0.001**
Problems related to negative life events	14	0.4%	142	0.7%	156	0.6%	**0.048**
Obsessive compulsive disorders	20	0.6%	69	0.3%	89	0.4%	**0.033**
Problems related to housing and economic circumstances	9	0.3%	62	0.3%	71	0.3%	0.656
Problems related to physical or social environment	8	0.2%	28	0.1%	36	0.1%	0.190
Induced psychotic disorders	7	0.2%	21	0.1%	28	0.1%	0.116
Hypnotic medication	N	%	N	%	N	%	
ALIMEMAZINE	–	–	6307	30.4%	–	–	
ZOPICLONE	–	–	5894	28.4%	–	–	
MELATONIN	–	–	1544	7.5%	–	–	
ZOLPIDEM	–	–	86	0.4%	–	–	
Number of hypnotics administered per stay	N	%	N	%	N	%	
None	–	–	10,260	49.5%	–	–	
One	–	–	7379	35.6%	–	–	
Two or more	–	–	3082	14.9%	–	–	

*Note:* The bold text represents the statistically significant results (*p*<0.05)

There was no significant difference between the two groups in the proportion of hospitalizations for nonpsychiatric causes, or related to social and economic difficulties. Notably, the prevalence of hospital stays related to cannabis use disorders was similar in both groups.

### Hypnotic use

**Figure 2.**
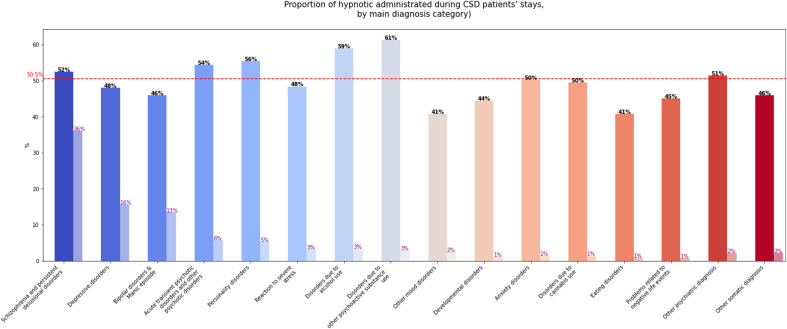
Proportion of hypnotic administered during stays of patients with chronic sleep disorders, by main psychiatric disorder. *Note*: The red dashed line represents the average proportion of stays during which a hypnotic was administered. The first bar represents the proportion of stays in which a hypnotic was administered for each psychiatric disorder, and the second bar (lighter one) represents the proportion of stays related to each psychiatric disorder relative to the total of stays. For instance, a hypnotic drug was administered in 52% of stays for schizophrenia and persistent delusional disorders, and those disorders represented 36% of all stays.

In the CSD group, a hypnotic medication was administered in 50.5% of hospital stays (Table 2). The most common molecule was alimemazine (30.4%), followed by zopiclone (28.4%), with more than one hypnotic administered in 14.9% of stays. Hypnotics were most frequently dispensed during stays related to psychoactive substance use disorders: 61% for substances other than alcohol or cannabis and 59% for alcohol use disorders. They were also administered in more than half of stays for personality disorders, acute and transient delusional disorders, and schizophrenia and persistent delusional disorders. In contrast, hypnotic prescriptions were lower for stays associated with mood and eating disorders (41% in each category). Figure 2 illustrates hypnotic use by primary diagnosis categories.

### Comorbidities

Patients in the NSD group had fewer comorbidities than patients in CSD group, as no comorbidity was reported in 71.1% of stays in NSD patients and 64.9% in CSD patients (p < 0.001). Patients in the CSD group more frequently presented two or more comorbidities (10.4% vs. 7.4%), regarding both psychiatric and other medical comorbidities (Table 3). Compared to NSD patients, those in the CSD group presented more often disorders due to psychoactive substance use (11.4% vs. 7.8%, p < 0.001), depressive disorders (3.5% vs. 2.6%, p = 0.005), a history of self-harm (1.8% vs. 1.3%, p = 0.044), reactions to severe stress (1.6% vs. 1.1%, p = 0.039), bipolar disorders (1.2% vs. 0.7%, p = 0.009), manic episodes (0.3% vs. 0.1%, p = 0.036), environmental or psychosocial difficulties (3.1% vs. 2.1%, p = 0.001), and functional intestinal disorders (2.6% vs. 1.3%, p < 0.001). Conversely, anxiety disorders were significantly more prevalent in NSD patients than in CSD patients (2.0% vs. 1.3%, p = 0.001).

**Table 3.** Comorbiditie**s**

	No sleep disorders (NSD) group (n = 3 524)		Chronic sleep disorders (CSD) group (n = 20 721)		All patients (n = 24 245)		
Number of comorbidities	N	%	N	%	N	%	*p*-value
No comorbidity	2506	71.1%	13458	64.9%	15964	65.8%	**<0.001**
One comorbidity	757	21.5%	5112	24.7%	5869	24.2%	
Two or more comorbidities	261	7.4%	2151	10.4%	2412	9.9%	
At least one psychiatric comorbidity	779	22.1%	5694	27.5%	6473	26.7%	**<0.001**
At least one nonpsychiatric comorbidity	166	4.7%	1220	5.9%	1386	5.7%	**0.005**
Prevalence by comorbidity	N	%	N	%	N	%	*p*-value
Disorders due to psychoactive substance use	275	7.8%	2360	11.4%	2635	10.9%	**<0.001**
Personality disorders	149	4.2%	963	4.6%	1112	4.6%	0.271
Schizophrenia, schizotypal, and delusional disorders	142	4.0%	873	4.2%	1015	4.2%	0.615
Problems related to housing and economic circumstances	150	4.3%	838	4.0%	988	4.1%	0.556
Depressive disorders	90	2.6%	721	3.5%	811	3.3%	**0.005**
Problems related to physical or social environment	74	2.1%	649	3.1%	723	3.0%	**0.001**
Functional intestine disorders	45	1.3%	532	2.6%	577	2.4%	**<0.001**
Essential (primary) hypertension	76	2.2%	433	2.1%	509	2.1%	0.798
Suicidal ideation	56	1.6%	403	1.9%	459	1.9%	0.152
Self-harm	46	1.3%	369	1.8%	415	1.7%	0.044
Diabetes	47	1.3%	335	1.6%	382	1.6%	0.212
Reaction to severe stress	40	1.1%	331	1.6%	371	1.5%	**0.039**
Resistance to drugs	17	0.5%	325	1.6%	342	1.4%	**<0.001**
Bipolar disorders	25	0.7%	251	1.2%	276	1.1%	**0.009**
Anxiety disorders	70	2.0%	269	1.3%	339	1.4%	**0.001**
Developmental disorders	19	0.5%	170	0.8%	189	0.8%	0.079
Eating disorders	25	0.7%	144	0.7%	169	0.7%	0.924
Obesity	16	0.5%	139	0.7%	155	0.6%	0.136
Manic episode	3	0.1%	57	0.3%	60	0.2%	**0.036**
Induced psychotic disorders	0	0.0%	7	0.0%	7	0.0%	0.603

*Note:* The bold text represents the statistically significant results (*p*<0.05)

## Discussion

This large-scale study of over 13,000 psychiatric inpatients reveals that CSD affects the vast majority (81%) of hospitalized patients, confirming their high prevalence in real-world psychiatric care. Importantly, CSD is not only common but also associated with more severe clinical profiles and a greater hospitalization burden, including increased frequency of hospitalizations, higher use and longer duration of seclusion, and more frequent physical restraint use. These findings highlight the considerable impact of sleep disturbances on inpatient care complexity and underscore the need to treat sleep not as a secondary symptom but as a core clinical dimension.

Patients with CSD had higher rates of depressive and bipolar disorders, personality disorders, and substance use disorders, especially those related to alcohol and other psychoactive substances. These associations align with existing literature linking sleep disruptions to affective instability, emotion dysregulation, and impulsivity [16, 34, 35]. Notably, hypnotics were prescribed in over half of the CSD-related stays, most commonly alimemazine and zopiclone, particularly in cases involving substance use and personality disorders. This pattern may reflect both the high clinical demand for sedation and the limited availability of nonpharmacological interventions such as cognitive behavioral therapy for insomnia (CBT-I) in inpatient settings, despite their efficacy as a first-line treatment, even during acute states [36, 37]. Beyond acute care, CBT-I has also been shown to reduce the risk of incident and recurrent depression. In a large randomized controlled trial, CBT-I decreased the risk of major depressive episodes over 36 months, particularly when sustained improvements in sleep were achieved [38]. These findings highlight the importance of addressing CSD not only to improve current symptoms but also to prevent relapse and recurrence of psychiatric disorders.

Patients with CSD also exhibited significantly more psychiatric and nonpsychiatric comorbidities, consistent with findings from previous studies, including prospective research [6]. The increased prevalence of conditions such as self-harm, depressive and bipolar comorbidities, stress-related disorders, environmental difficulties, and functional intestinal disorders points to a more complex and multidimensional clinical profile in patients with psychiatric disorders and comorbid CSD. Indeed, the dysregulation of sleep–wake rhythms and sleep disorders, beyond their negative impact on emotional regulation, can lead to dysfunction of the autonomic nervous system, impairment of the hypothalamic–pituitary–adrenal axis, and immune dysregulation [39]. These findings support the hypothesis that sleep disturbances are not isolated symptoms, but are integrated components of broader vulnerability profiles in psychiatric disorders.

Unexpectedly, some conditions – including schizophrenia, acute psychotic episodes, neurodevelopmental disorders, eating disorders, and anxiety disorders – were more common in patients without identified sleep disorders (NSD group). Several hypotheses may explain this counterintuitive finding. First, patients with severe cognitive or psychotic symptoms may be less likely to report sleep complaints, leading to underrecognition [40]. Second, clinicians may focus on acute psychotic symptoms during hospitalization, deprioritizing the assessment or documentation of sleep. Moreover, sleep disturbances are part of the diagnostic criteria for mood disorders, such as depression and bipolar disorder, but not for psychotic disorders. This raises the question of whether these symptoms are systematically assessed in patients with psychotic disorders. Also, sleep disorders may be underestimated in neurodevelopmental and eating disorder populations, where symptom expression is often atypical. Additionally, the CSD classification relied partly on documented evidence, such as medical record notes and hypnotic prescriptions, which may be less consistently recorded in certain diagnostic groups.

Also, previous studies have also highlighted the impact of hospitalization itself on sleep disturbances, both in terms of sleep quality and duration. Although these studies focused on hospitalizations for nonpsychiatric reasons, they emphasized that changes in daily rhythms and disruptive factors such as noise can impair sleep quality during the hospital stay – underlining the importance of addressing sleep-related symptoms in already vulnerable patients [41].

These results open important clinical perspectives. We anticipated a high prevalence of CSDs in psychiatric inpatients, but the magnitude observed (81%) and their strong associations with hospitalization burden and restraint use were greater than expected. These findings highlight the need for systematic and standardized assessment of sleep disorders in all hospitalized patients with psychiatric disorders, particularly in those with communication difficulties or during acute episodes. The high rate of hypnotic prescription – especially in cases involving sedative use for behavior management – also calls for careful consideration of pharmacological strategies, and where possible, greater integration of nondrug approaches, such as CBT-I or chronotherapy [36, 42, 43]. Efforts such as the Delphi-based validation of a standardized assessment set for sleep, chronobiology, addictive, and psychiatric dimensions in the SoPsy-Depression French national cohort illustrate the feasibility and importance of implementing structured, consensus-based tools in clinical and research settings to better characterize and treat sleep disturbances in psychiatric populations [44]. Future research should validate our operational definition of CSD in independent cohorts, and develop standardized screening tools for routine practice. Interventional studies are also warranted to determine whether targeted sleep management can reduce hospitalization burden, improve quality of life, and lower relapse rates.

Several limitations should be acknowledged. First, the study relied on routinely collected clinical data, which may result in underreporting or inconsistent documentation of sleep complaints. Second, ICD-10 coding and free-text analyses, while comprehensive, may not fully capture sleep disorders diagnosed outside standardized classifications (e.g., insomnia symptoms not coded explicitly). Third, the population was limited to inpatients during acute episodes, which may restrict generalizability to community or outpatient settings. Finally, while the ILSD threshold (>0.5) provides an innovative operational definition of chronicity, it requires further validation in other cohorts.

Despite these limitations, this study offers important real-world insights into the clinical burden and characteristics of CSDs in psychiatric hospital settings. By demonstrating the links between CSD, severe psychiatric presentations, increased restraint use, and polypharmacy, the findings underscore the urgent need to integrate sleep-focused evaluation and interventions as part of comprehensive psychiatric care.

## Conclusion

In conclusion, CSDs are not only highly prevalent in psychiatric inpatient care but are also associated with increased clinical burden and complex comorbidity profiles. Recognizing and treating sleep disturbances as core components of psychiatric disorders – not as secondary symptoms – is essential for improving patient outcomes and quality of care.

## Data Availability

Data supporting the findings of this study are derived from the Paris Psychiatry and Neurosciences Hospital Group health data warehouse. Due to French data protection regulations and patient confidentiality, individual-level data cannot be shared publicly. Access to aggregated or anonymized data may be granted upon reasonable request and with approval from the institutional Data Access Committee.
